# Natural History of Cardiac and Respiratory Involvement, Prognosis and Predictive Factors for Long-Term Survival in Adult Patients with Limb Girdle Muscular Dystrophies Type 2C and 2D

**DOI:** 10.1371/journal.pone.0153095

**Published:** 2016-04-27

**Authors:** Abdallah Fayssoil, Adam Ogna, Cendrine Chaffaut, Sylvie Chevret, Raquel Guimarães-Costa, France Leturcq, Karim Wahbi, Helene Prigent, Frederic Lofaso, Olivier Nardi, Bernard Clair, Anthony Behin, Tanya Stojkovic, Pascal Laforet, David Orlikowski, Djillali Annane

**Affiliations:** 1 Service de Réanimation médicale et unité de ventilation à domicile, centre de référence neuromusculaire GNHM, CHU Raymond Poincaré, APHP, Université de Versailles Saint Quentin en Yvelines, Garches, France; 2 Centre d’Investigation clinique et Innovation technologique CIC 14.29, INSERM, Garches, France; 3 Institut de Myologie, CHU Pitié Salpetrière, Centre de référence neuro musculaire Paris Est, Université Pierre et Marie Curie Paris VI, Paris, France; 4 SBIM, CHU Saint Louis, APHP, Université Paris Diderot, Paris, France; 5 Service de Physiologie - Exploration fonctionnelles, CHU Raymond Poincaré, APHP, Université de Versailles saint Quentin en Yvelines, Garches, France; 6 Service de cardiologie, Hôpital Cochin, APHP, Université Paris Descartes-Sorbonne Paris Cité, Paris, France; 7 Laboratoire de biochimie et génétique moléculaire, hôpital Cochin, AP-HP, université Paris Descartes-Sorbonne Paris Cité, Paris, France; Cincinnati Children's Hospital Medical Center, UNITED STATES

## Abstract

**Background:**

Type 2C and 2D limb girdle muscular dystrophies (LGMD) are a group of autosomal recessive limb girdle muscular dystrophies manifested by proximal myopathy, impaired respiratory muscle function and cardiomyopathy. The correlation and the prognostic impact of respiratory and heart impairment are poorly described. We aimed to describe the long-term cardiac and respiratory follow-up of these patients and to determine predictive factors of cardio-respiratory events and mortality in LGMD 2C and 2D.

**Methods:**

We reviewed the charts of 34 LGMD patients, followed from 2005 to 2015, to obtain echocardiographic, respiratory function and sleep recording data. We considered respiratory events (acute respiratory failure, pulmonary sepsis, atelectasis or pneumothorax), cardiac events (acute heart failure, significant cardiac arrhythmia or conduction block, ischemic stroke) and mortality as outcomes of interest for the present analysis.

**Results:**

A total of 21 patients had type 2C LGMD and 13 patients had type 2D. Median age was 30 years [IQR 24–38]. At baseline, median pulmonary vital capacity (VC) was 31% of predicted value [20–40]. Median maximal inspiratory pressure (MIP) was 31 cmH_2_O [IQR 20.25–39.75]. Median maximal expiratory pressure (MEP) was 30 cm H_2_O [20–36]. Median left ventricular ejection fraction (LVEF) was 55% [45–64] with 38% of patients with LVEF <50%. Over a median follow-up of 6 years, we observed 38% respiratory events, 14% cardiac events and 20% mortality. Among baseline characteristics, LVEF and left ventricular end diastolic diameter (LVEDD) were associated with mortality, whilst respiratory parameters (VC, MIP, MEP) and the need for home mechanical ventilation (HMV) were associated with respiratory events.

**Conclusion:**

In our cohort of severely respiratory impaired type 2C and 2D LGMD, respiratory morbidity was high. Cardiac dysfunction was frequent in particular in LGMD 2C and had an impact on long-term mortality.

**Trial Registration:**

ClinicalTrials.gov NCT02501083

## Introduction

Type 2C and 2D limb girdle muscular dystrophies (LGMD) are autosomal recessive muscular dystrophies that belong to the group of sarcoglycanopathies (SG). They are characterized by mutations in the genes encoding sarcoglycans. Mutations in *gamma*-sarcoglycan (SGCC gene) [1] and *alpha*-sarcoglycan (SGCA gene) [2] are responsible for LGMD 2C and LGMD 2D respectively. Sarcoglycans belong to a group of proteins associated with dystrophin (dystrophin associated glycoprotein, DAG). The DAG complex gives structural stability to the muscle fiber, linking dystrophin with the plasma membrane of muscle cells and the extracellular matrix [3]. The loss or reduction of sarcoglycans’ function results in the destabilization of the DAG complex, and may be related to development of muscular dystrophy. The related impact on respiratory function and the presence of cardiomyopathy are associated with reduced life expectancy, particularly in Duchenne muscular dystrophy (DMD) [4] where respiratory failure is a leading cause of morbidity and mortality [5]. Sleep-related breathing disorders (SRBD) have also frequently been observed in DMD and may affect disease prognosis [6,7].

Clinical presentation of LGMD is diverse but usually shows predominant proximal weakness. Cardiac involvement, ranging from electrocardiographic (ECG) abnormalities to dilated cardiomyopathy (DCM), has been reported in 17% to 50% of patients with sarcoglycanopathies (SG) [8,9]. The involvement of striated muscles may also result in an impaired respiratory muscle function (10). Patients with 2C and 2D LGMD seem to have a greater respiratory impairment than other sarcoglycanopathies [10]. Long-term noninvasive ventilation (NIV) has resulted in improved survival for patients with muscular dystrophy [11]. However, the evolution of respiratory and heart function in muscular dystrophy patients, as well as their respective impact on prognosis, has been poorly described.

The aims of the study conducted in a group of 34 LGMD 2C and 2D patients followed-up for a maximum of 10 years were to describe the long-term follow-up of their cardiac and respiratory function, to assess the eventual association between cardiac and respiratory impairment and to establish long-term survival rate and predictive factors of cardiac and respiratory events and of mortality in 2C and 2D LGMD.

## Methods

### Study design

We retrospectively reviewed the charts of all LGMD 2C and 2D followed at the Home Mechanical Ventilation Unit of the Raymond Poincare University Hospital, a tertiary neuromuscular center (Garches, France). Neuromuscular patients are seen in the unit at least yearly to monitor their respiratory function.

We included all adult patients (>18 years) with genetically confirmed 2C or 2D LGMD. These patients were addressed to the unit for assessment and management of respiratory function because of a suspected respiratory impairment. For each patient, we collected echocardiographic results, respiratory function, sleep recording and outcome data. The initial visit in the unit that includes both a respiratory and an echocardiographic assessment was considered the baseline for the present study.

The study was performed in compliance with the ethical principles formulated in the declaration of Helsinki and was approved by the *comité de protection des personnes* and the *commission nationale de l'informatique et des libertés*. The study was registered in ClinicalTrials. gov (identifier: NCT02501083).

### Genetic tests

*SGCA* and *SGCC* gene analyses for mutation identification were performed using direct sequencing or multiplex quantitative fluorescent PCR (QF-PCR) of genomic DNA extracted from whole blood using standard procedures.

### Cardiac function

Echocardiography was performed with a Siemens CV70 device. Echocardiographic measurements were made and interpreted according to the guidelines published by the American Society of Echocardiography [12] by two experienced cardiologists (AF and ON). Patients with LVEF <50% were considered to have left ventricular systolic dysfunction [13].

### Respiratory Function and Nocturnal Sleep Recording

A pulmonary function testing system (Vmax 229, SensorMedics, Yorba Linda, California) was used to make the spirometric-static measurements of vital capacity (VC), according to standard guidelines [14]. For the measurement of maximal inspiratory pressure (MIP) at the residual volume and maximal expiratory pressure (MEP) at total lung capacity, patients breathed into a mouthpiece connected to a manometer. The maneuvers were repeated at least three times or until two identical readings were obtained. For each maneuver, the best result was kept for the study [15].

From the routinely performed daytime arterial or arterialized capillary blood gases, we recorded diurnal carbon dioxide tension (pCO2) and bicarbonate. The results of nocturnal oximetry, performed with a Covidien Nellcor oximeter were also collected (percentage of sleep time with oxygen saturation SaO2 <90%). We also recorded apnea hypopnea index (AHI) from the first available polysomnography after the initial visit.

### Outcomes

We considered respiratory events, cardiac events and mortality as outcomes of interest for the present analysis. Cardiac events were defined as the onset of either acute heart failure syndrome (AHF), cardiac arrhythmia (atrial fibrillation, atrial flutter, ventricular tachycardia, and ventricular fibrillation), significant conduction block (atrioventricular block type II or III, sino atrial block type III) or ischemic stroke. The diagnosis of arrhythmia or conduction block was confirmed by a cardiologist (AF or ON) reviewing the original documents.

Respiratory events were defined as the onset of pulmonary sepsis, acute respiratory failure (ARF), atelectasis or pneumothorax.

For each outcome, only the first occurrence was considered. As a part of the routine follow-up, neuromuscular disease patients are contacted at least annually to plan their follow-up visit in our unit and to record vital status. During each visit, details of intercurrent hospitalizations are systematically collected in the medical charts.

### Statistical Analysis

Continuous variables were described by median ± interquartile range (IQR) and compared by Wilcoxon Rank Sum test; dichotomous or categorical variables were described by number of subjects and percentage and compared by Fisher’s exact test. The associations between respiratory and cardiac continuous parameters were explored by the nonparametric Spearman’s correlation coefficient. Survival curves were estimated by the Kaplan-Meier method and then compared by the log-rank test; univariable Cox models allowed to estimate the strength of association based on the hazard ratio (HR). Statistical analysis was performed using R^®^ (http://www.r-project.org/).

## Results

A total of 34 patients were included in our study: 21 (62%) had LGMD 2C and 13 (38%) had LGMD 2D.

Age ranged from 20 years to 48 years with a median age of 30 years; median age of ambulation loss was 15 years [14–21]. Baseline characteristics of the population are detailed in Table 1 (percentages were calculated separately in each group of patients).

**Table 1:** Characteristics of the study population.

	All	LGMD 2D	LGMD 2C	P value
N	34 (100%)	13 (38.23%)	21 (61.77%)	
Age (y)	30 [24; 38]	30 [25; 40]	27 [23; 37]	0.27
Male gender	20 (58.82%)	7 (53.85%)	13 (61.9%)	0.73
Age of ambulation loss (y)	15 [14; 21]	15 [14; 20.25]	15 [13; 21]	0.50
Pre-albumin (mg/dl)	0.24 [0.22; 0.33]	0.23 [0.22; 0.33]	0.25 [0.22; 0.32]	0.95
***Respiratory function***				
VC (ml)	1140 [850; 1820]	1110 [530; 1500]	1175 [850; 1842]	0.33
VC (% pred)	31 [20; 39.75]	28 [19; 40]	33 [23; 39]	0.62
MIP (cmH2O)	31 [20.25; 39.75]	27 [11.25; 41.5]	33.5 [21.75; 38.25]	0.81
MEP (cmH2O)	30.5 [20.25; 35.75]	30.5 [23.75; 42.75]	30.5 [19.75; 35.25]	0.77
HMV	12 (35.29%)	5 (38.46%)	7 (33.33%)	1.00
Tracheostomy	3 (8.82%)	1 (7.69%)	2 (9.52%)	1.00
Diurnal pCO2 (kPa)	5.75 [5.42; 6.27]	5.7 [5.34; 6.05]	5.75 [5.57; 6.34]	0.36
Diurnal bicarbonates (mmol/l)	26.9 [25.4; 28.6]	26.7 [25.75; 28.45]	26.9 [25.4; 28.9]	0.96
AHI	7.7 [3.5; 19]	11.4 [6.2; 15.2]	4 [4; 19]	0.88
Nocturnal SatO2<90%—(% of the recording time)	1 [0; 1.5]	1 [0; 1]	1 [1; 1.87]	0.26
***Cardiac function***				
LVEF (%)	55 [45; 64]	63 [57; 65]	48.5 [42.25; 58.5]	0.014
LVEDD (mm)	41.5 [37.25; 47.25]	39[38; 42]	44 [37; 49]	0.37
ACE inhibitors	13(38.24%)	2 (15.38%)	11 (52.38%)	0.06
Beta-blockers	10 (29.41%)	0 (0%)	10 (47.62%)	
Sinus rhythm	34 (100%)	13 (100%)	21 (100%)	
HR (bpm)	88.5 [80; 93.75]	92 [86.75; 94.5]	86 [76.25; 90.5]	0.13

Values are expressed ad median [interquartile range] or number (proportion).

VC: pulmonary vital capacity (% pred: percent of the predicted value)

MIP: maximal inspiratory pressure

MEP: maximal expiratory pressure

HMV: home mechanical ventilation

AHI: apnea hypopnea index

SatO2: percentage of oxygen saturation

LVEF: left ventricular ejection fraction

LVEDD: left ventricular end diastolic diameter

ACE: angiotensin-converting enzyme

HR: heart rate

### SGCA and SGCC Gene Analysis

Two SGCC mutations were found. All patients with LGMD 2C had the homozygous mutation Del525T in exon 6 except 2 patients who were carrying the homozygous mutation C283Y in exon 8. The following mutations were found in LGMD 2D patients (gene SGCA): homozygous R77C (4/14 patients, homozygous IVS1+3A> T (3/14 patients), R77C/K252Vfs*8 (2/14 patients), R98H and V75A (2 patients/14), R77C+R74W (1 patient), R98H/IVS7-1G> T (1 patient).

### Respiratory and Cardiac Function

Included patients had severe restrictive respiratory defects with a median [IQR] pulmonary vital capacity (VC) of 31% [20–40] of predicted capacity. About 35% of the patients were on home mechanical ventilation at the initial visit, mostly non-invasive (3 patients ventilated by tracheostomy) (Table 1). A total of 13 out of 34 patients (38%) had left ventricular systolic dysfunction. LV dysfunction was mild (LVEF 40–50%) in 20.5% of patients, moderate (LVEF 30–40%) in 8.8% and severe (LVEF<30%) in 8.8% of patients.

When comparing patients with 2C and 2D LGMD, we observed no differences in respiratory function, whereas LGMD 2C patients showed more severe cardiac impairment (Table 1). We found no significant association between respiratory and cardiac function at baseline (p = 0.76 for the correlation between LVEF and VC).

**Fig 1:**
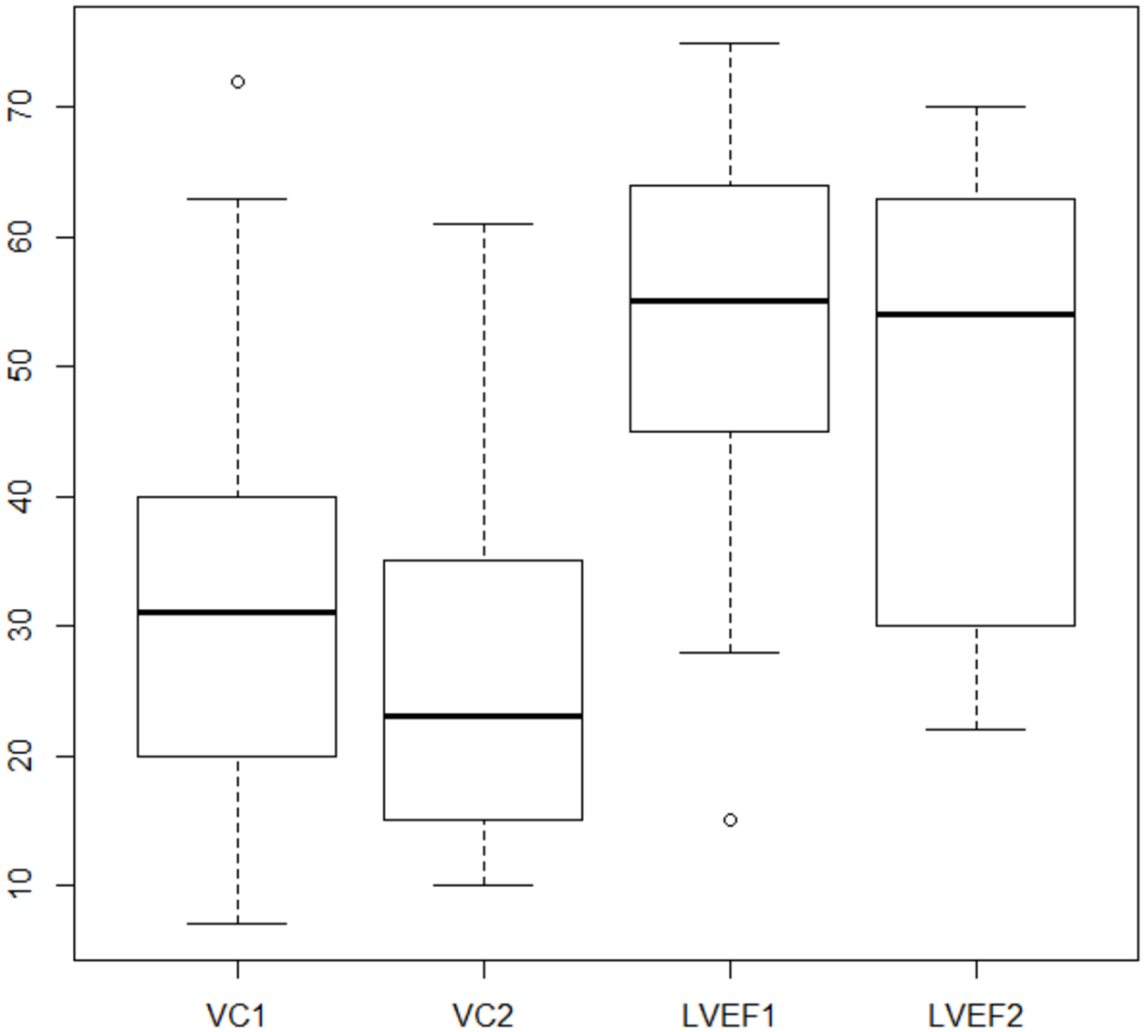
Evolution of respiratory vital capacity (VC) and left ventricular ejection fraction (LVEF) over the follow-up time. Follow up time was 65.6 [IQR 46.28; 80.68] months between VC1 and VC2 and 80.8 months [IQR 53.1; 89] months between LVEF1 and LVEF2. VC: pulmonary vital capacity. LVEF: left ventricular ejection fraction.

A follow-up evaluation of LVEF was available for 18 patients after a median time of 77.8 months [IQR 49.2–88.7] months and showed a stable cardiac function over time. On the other hand, respiratory function deteriorated during the 65.6 months [IQR 46.3–80.7] follow-up for the 24 patients for which this information was available (Fig 1).

Twelve patients started HMV during their follow-up. At the end of the follow-up period, 24 patients (70%) were treated by HMV.

### Apnea Hypopnea Index

AHI was available only in 8 patients raging from 1 to 38 without any differences in the 2 groups (p 0.88) (Table 1).

### Long-term Outcomes and Predictors of Outcomes

**Fig 2:**
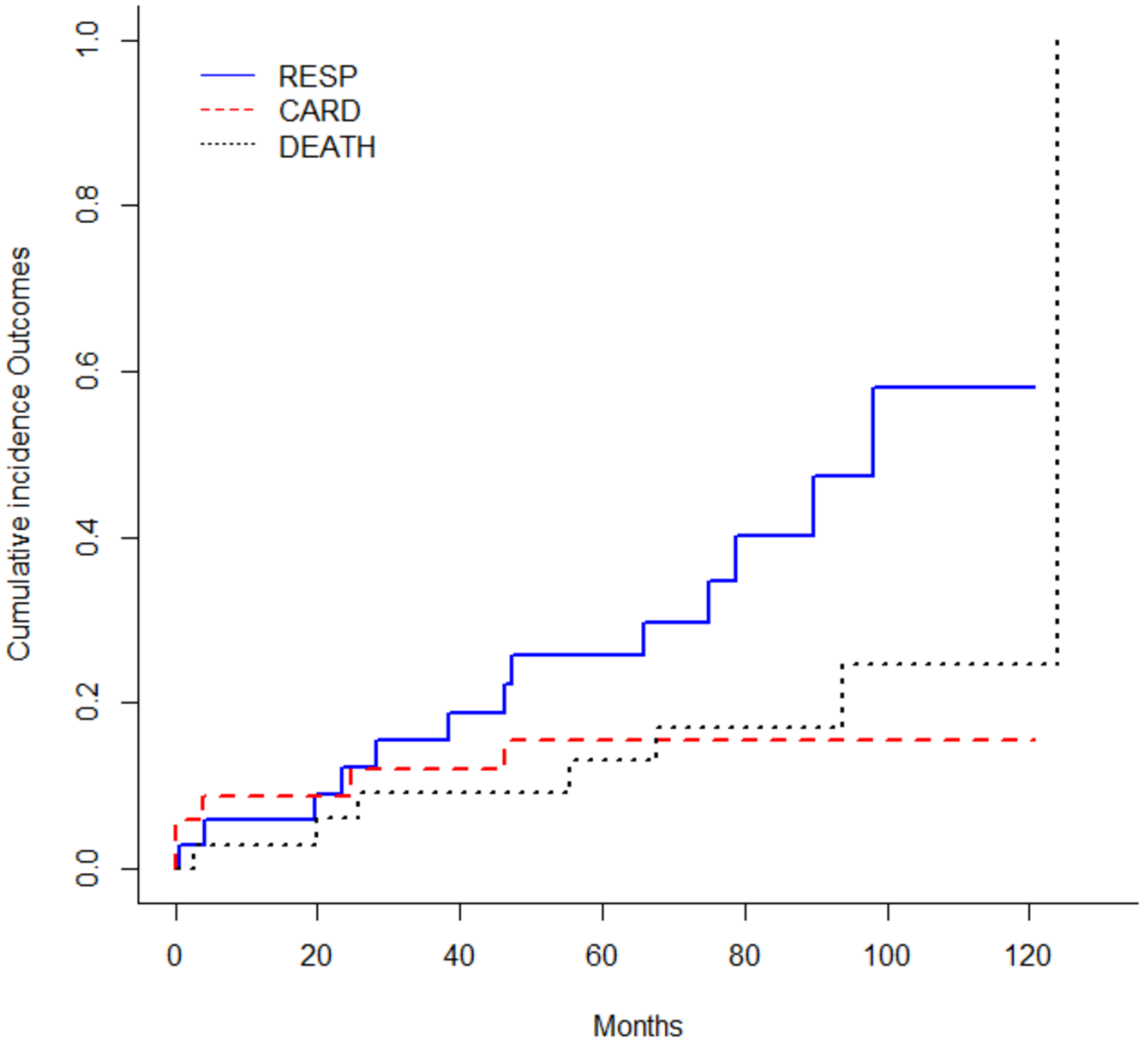
Cumulative incidence of the studied outcomes.

Over a median follow-up of 6 years [IQR 41–96 months], we observed 13 respiratory events (38%), 5 cardiac events (15%) and 7 deaths (21%). A total of 16 patients (47%) experienced at least one of the 3 outcomes. The cumulative incidence of these outcomes during the follow up time is represented in Fig 2.

**Fig 3:**
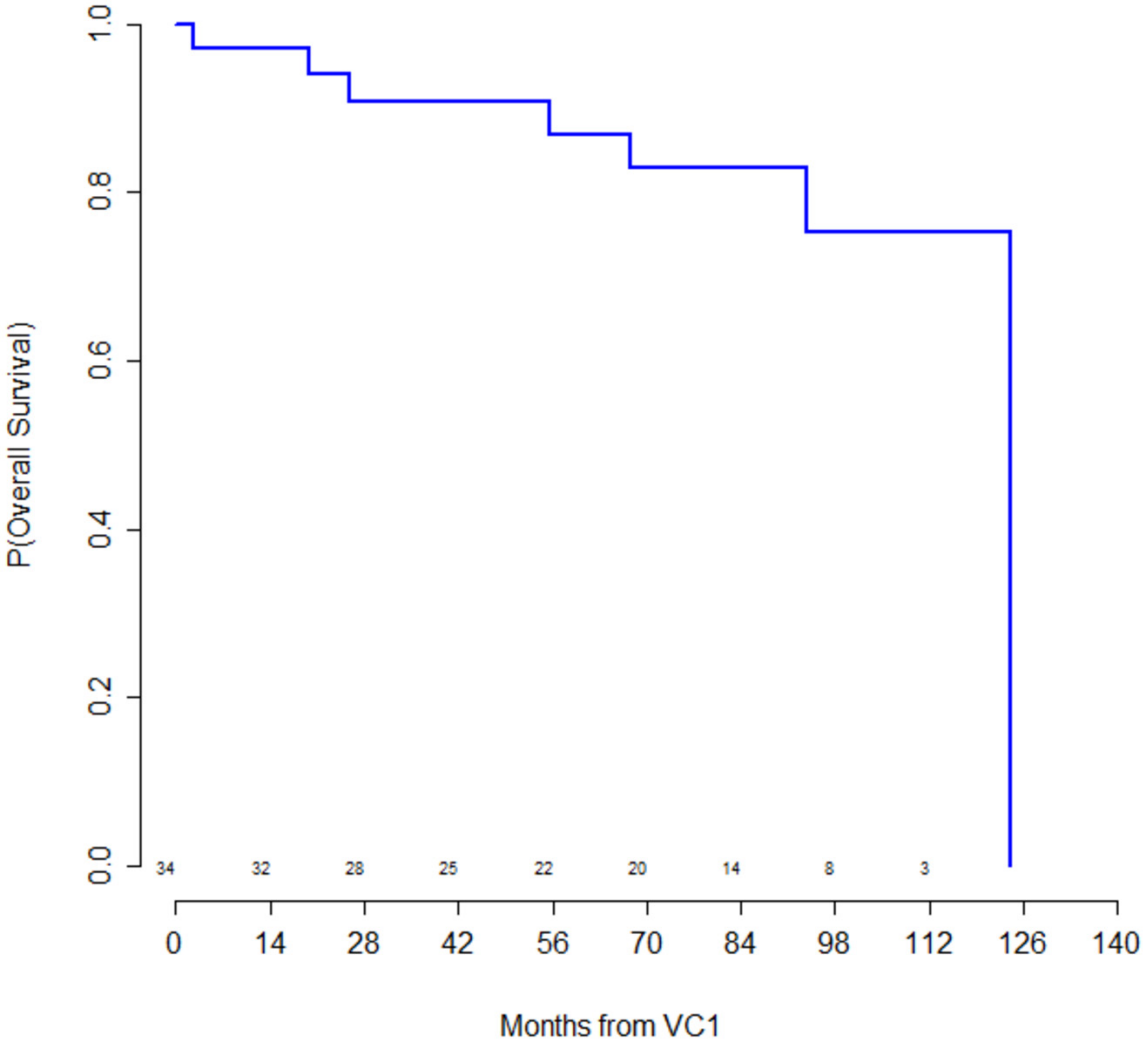
Global survival of the study population.

The Fig 3 shows the global survival curves of patients with LGM2D and 2C over time.

Among respiratory events, 13 patients (38%) experienced acute respiratory failure (ARF), 7 (20%) patients had pulmonary sepsis, 1 patient had atelectasis and 1 patient had pneumothorax.

Among cardiac events, 4 patients (12%) had acute heart failure (AHF) and 2 patients had ischemic stroke (6%). Respiratory parameters at baseline were significantly associated with the occurrence of respiratory events, with an OR of 0.93 (95% CI 0.87–0.99, p 0.008) for VC and an OR 6.80 (95% CI 1.4–32.4, p 0.025) for the need for HMV (Table 2). MIP and MEP were significantly associated with the occurrence of respiratory events with an OR of 0.93 (95%CI 0.87–1.00) for MIP and an OR 0.94 (95% CI 0.88–1.01) for MEP (Table 2).

**Table 2:** Factors associated with respiratory events.

Factors	No respiratory events (N = 21)	Respiratory events(N = 13)	P value
LGM 2D	33.33%	46.15%	0.49
Age (y)	25 [23; 38]	31 [30; 38]	0.17
Male gender	61.9%	53.85%	0.73
Age of ambulation loss (y)	14.5 [13.25; 20.5]	16 [14; 20.5]	0.83
Pre albumin (mg/dl)	0.24 [0.22; 0.31]	0.3 [0.22; 0.33]	0.50
Baseline VC (ml)	1630 [1125; 1985]	850 [590; 1010]	0.004
Baseline VC (%pred)	35 [26; 43]	20 [16; 28]	0.008
MIP (cmH2O)	37 [23; 40.5]	22 [14; 29.5]	0.047
MEP (cmH2O)	33 [23.5; 37.5]	25 [15; 30.5]	0.055
Diurnal pCO2 (kPa)	5.6 [4.97; 5.97]	6.2 [5.65;6.32]	0.08
Diurnal bicarbonate (mmol/l)	26.3 [25; 28.1]	28.3 [25.9; 29.6]	0.069
Nocturnal SatO2<90% (%)	1 [0; 1.25]	1 [0.25; 1.75]	0.77
HMV	19.05%	61.54%	0.025
Tracheostomy	4.76%	15.38%	0.54
LVEF (%)	55 [45; 64.25]	55[45; 64]	0.88
LVEDD (mm)	43 [38.25; 48]	40.5 [37; 45]	0.64

Values are expressed ad median [interquartile range] or number (proportion).

VC: pulmonary vital capacity

MIP: maximal inspiratory pressure

MEP: maximal expiratory pressure

SatO2: percentage of oxygen saturation

HMV: home mechanical ventilation

HR: heart rate

LVEF: left ventricular ejection fraction

LVEDD: left ventricular end diastolic diameter

The level of diurnal pCO2 tended to be associated with the occurrence of respiratory events with an OR of 2.91 (95%CI 0.85–9.91). Diurnal bicarbonate level tended to be significantly associated with the occurrence of respiratory events with an OR of 1.41 (95% CI 0.99–2.00).

On the other hand, cardiac events were associated with LVEF at baseline (p 0.004), but not with respiratory parameters, excepted for the presence of tracheostomy (Table 3). Baseline HR did not predict the occurrence of cardiac events (Table 3).

**Table 3:** Factors associated with cardiac events.

Factors	No cardiac events (n = 29)	Cardiac events (n = 5)	P value
LGM 2D	44.83%	0%	
Age (y)	30 [24; 39]	30 [23; 31]	0.53
Male gender (%)	41.38%	40%	1.00
Age of ambulation loss (y)	15 [14; 19]	18.5 [12.75; 23]	1.00
Pre-albumin (mg/dl)	0.24 [0.22; 0.33]	0.32 [0.23; 0.33]	1.00
Baseline VC (%pred)	1210 [850; 1820]	980 [690; 1358]	0.51
MIP (cmH2O)	33.5 [20.25; 40]	29.5 [24.25; 31.75]	0.52
MEP (cmH2O)	30.5 [21.25; 35.75]	26.5 [17; 37.5]	0.85
Diurnal pCO2 (kPa)	5.85 [5.5; 6.29]	5.2 [4.41;5.82]	0.20
Diurnal bicarb (mmol/l)	26.9 [25.7; 28.6]	23.8 [21; 26.8]	0.19
Nocturnal SatO2<90% (%)	1[0; 1.37]	1[1; 5.5]	0.27
HMV	34.48%	40%	1.00
Tracheostomy	3.45%	40%	0.050
HR (bpm)	88 [80; 94]	89 [88.5; 90]	0.89
LVEF (%)	59 [48; 65]	30 [28; 40]	0.004
LVEF <55% (%)	42.86%	100%	0.04
LVEDD (mm)	40.5 [37; 45.75]	52.5 [48.25; 56.75]	0.15

Values are expressed ad median [interquartile range] or number (proportion).

VC: pulmonary vital capacity

MIP: maximal inspiratory pressure

MEP: maximal expiratory pressure

SatO2: percentage of oxygen saturation

HMV: home mechanical ventilation

HR: heart rate

LVEF: left ventricular ejection fraction

LVEDD: left ventricular end diastolic diameter

**Fig 4:**
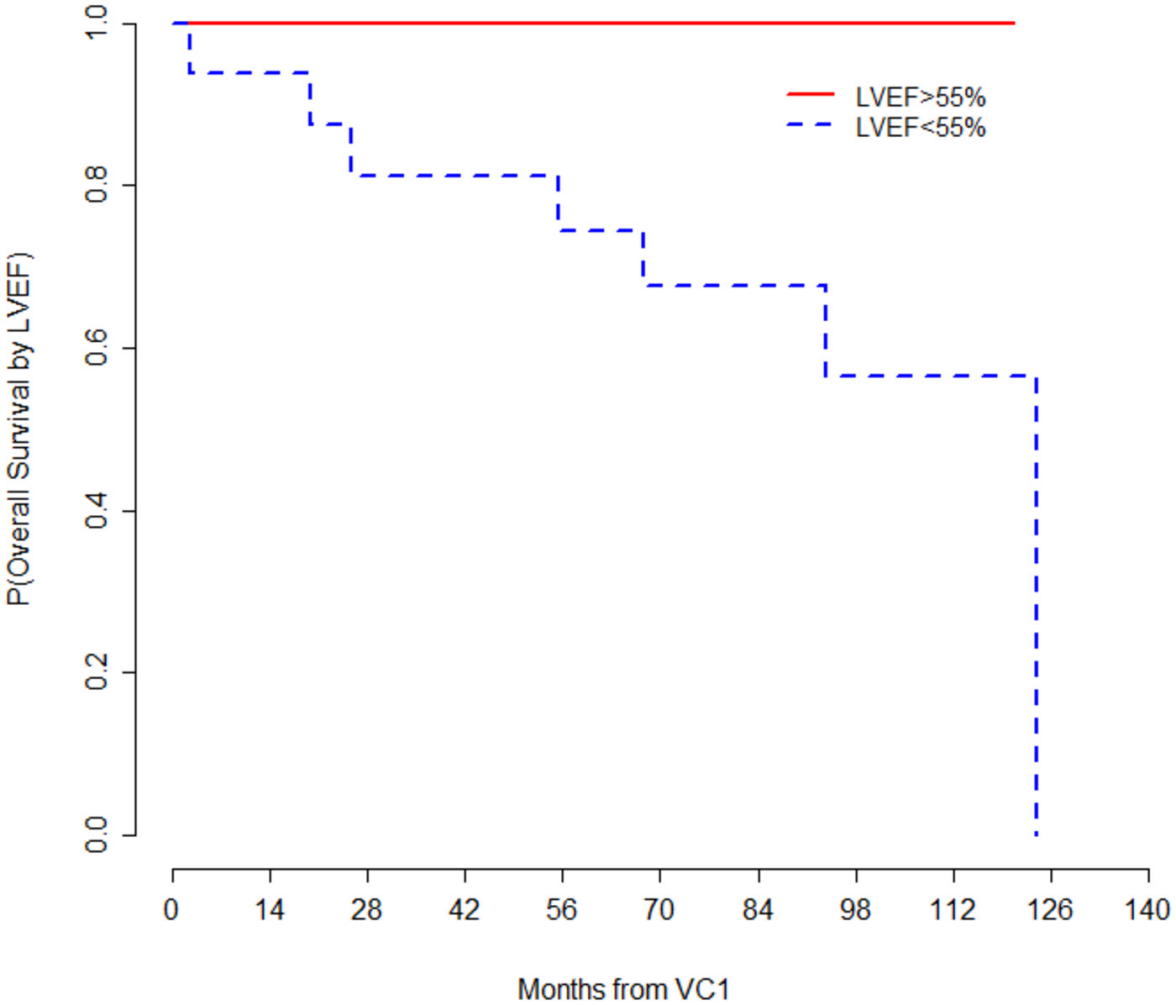
Mortality according to left ventricular function. Patients were divided in the two groups using the median value of LVEF (55%) as a cut-off.

Cardiac function at baseline with impaired LVEF (p 0.001) and left ventricular end diastolic diameter (LVEDD) were associated with mortality (p 0.05). No association was found between respiratory impairment and survival (Table 4 and Fig 4). We observed no differences in mortality between the types of sarcoglycanopathy.

**Table 4:** Factors associated with mortality.

Factors	Alive	Death	P value
LGM 2D	44.44%	14.29%	0.21
Age (y)	30 [24; 37.5]	31 [26.5; 41.5]	0.46
Male gender	55.56%	71.43%	0.67
Age of ambulation loss (y)	14.5 [13.75; 18.25]	23 [14; 23]	0.45
Pre albumin (mg/dl)	0.24 [0.22; 0.33]	0.33 [0.27; 0.37]	0.37
Baseline VC (% pred)	31 [21; 39.5]	35 [18; 40]	0.98
Baseline VC (ml)	1210[850; 1760]	1025 [842; 1718]	0.77
MIP (cmH2O)	33.5[20.75; 40.25]	29.5[14.75; 35.25]	0.36
MEP (cmH2O)	30.5 [20.5; 36.25]	30 [22.25; 32.5]	0.84
Diurnal pCO2 (kPa)	5.85 [5.5; 6.3]	5.2 [4.7; 5.8]	0.11
Diurnal bicarbonates (mmol/l)	26.9 [25.8; 28.5]	25.6 [22.6; 28.6]	0.35
Nocturnal SatO2<90% (%)	1 [0; 1]	1.25 [1; 3.6]	0.15
AHI	7.7 [2.5; 17.1]	11.5 [7.75; 15.25]	0.87
HMV	33.33%	42.86%	0.68
Tracheostomy	3.7%	28.57%	0.10
HR(bpm)	89 [80; 94]	84 [80; 89]	0.37
LVEF (%)	60 [49.25; 65]	40 [29; 45]	0.001
LVEF<55%	38.46%	100%	0.007
LVEDD(mm)	40[37; 44.5]	50 [47; 55.5]	0.055

Values are expressed ad median [interquartile range] or number (proportion).

VC: pulmonary vital capacity

MIP: maximal inspiratory pressure

MEP: maximal expiratory pressure

SatO2: percentage of oxygen saturation

HMV: home mechanical ventilation

HR: heart rate

LVEF: left ventricular ejection fraction

LVEDD: left ventricular end diastolic diameter

## Discussion

We describe the first available data on the association between cardiac and respiratory impairment in a cohort of 34 patients with 2C and 2D LGMD, followed in our reference center for a period of up to 10 years. We also described the evolution of their cardiac and respiratory function over time as well as the association between function reduction and disease prognosis.

### Heart

In this study, left ventricular function was altered in 38% of patients. Patients with mutations in *gamma* sarcoglycan had more severe cardiac dysfunction than patients with *alpha* sarcoglycan mutations. This can be partially explained by the consequences of the related mutations [16]. SGCA are missense that still leads to protein synthesis, thus maintaining some sarcoglycan function, whereas mutations in SGCC are usually truncation without protein production [17]. Our results confirm the preliminary results of a previous study by our team [18], which included 19 patients with *alpha* and *gamma* sarcoglycanopathies. Left ventricular systolic dysfunction was observed in 30% of patients. Heart involvement has been previously reported in a few series of patients with sarcoglycanopathies [9,10,19]. A high prevalence of cardiac impairment (29% and 67%) has been reported in patients with respectively LGMD 2I and LGMD 2E, without correlation with age or skeletal muscle strength [20]. In a second study, cardiac involvement was reported in 63% of patients with LGMD type 2E [21]. Preclinical cardiomyopathy was present in 44% of patients with sarcoglycanopathies in an Italian study, where 2/20 patients (10%) had an initial dilated cardiomyopathy [10]. In a recent Dutch study, 17% out of the 24 assessed patients with sarcoglycanopathy showed dilated cardiomyopathy [8]. In our study, we observed a higher prevalence than the previously published studies. This may be related to the differences in disease duration as suggested from the differences in the median ages: 23 years for the Italian study, 25 years in the Dutch study and 30 years in our population. Acute ischemic stroke was found in 6% of our patients and was related to dilated cardiomyopathy. Little is known about this complication in LGMD [22]. These results highlight the need for potential preventive treatment in LGMD patients with severe heart failure.

### Pulmonary and Sleep

The involvement of striated muscles impairment may result in reduced respiratory muscle function in patients with LGMD. Respiratory defects may be present early, particularly in LGMD 2I [23,24]. In our study all patients had a severe restrictive respiratory failure at the baseline visit, and 70% needed home mechanical ventilation at the end of the follow-up. This prevalence is remarkably higher than what described in the study by Politano *et al*. [10], in which a mild respiratory impairment was detected in 35% of patients and 12% showing severe respiratory failure. In the study by Poppe *et al*. [25], 36% of patients with LGMD 2I showed moderate respiratory impairment defined by a VC between 41% and 75%. Severe respiratory defects, defined by VC between 19% and 40%, was seen only in 8% of the patient despite a similar age profile than our population.

Major differences between the Italian and present study were the median age of the studied population, and the specificities of the recruitment centers: a cardiology department for the Italian study and a respiratory center for neuromuscular diseases in our study.

In our population, we found no association between the severity of the respiratory dysfunction and presence of cardiac defects. Not surprisingly, our study found a significant association between the level of MIP, the level of MEP and the occurrence of respiratory events in LGMD. Indeed, MIP and MEP explore respiratory muscle strength. With expiratory muscle failure, patients are at risk of respiratory sepsis and failure because of the impairment of cough and respiratory airway clearance.

Neuromuscular diseases may be associated with ventilatory disturbances and sleep-related breathing disorders (SRBD) [26]. SRBD may be present early in the natural history of patients with DMD and occurred in 64% of patients in the study by Suresh *et al*. [6]. A moderate decrease of VC in patients with LGMD has been reported by Gigliotti *et al*. [27] with a good correlation between daytime pCO2 and VC. Moreover, functional pulmonary tests may predict nocturnal hypoventilation [28,29]. In our study, we found few abnormal sleep recordings, which may be explained by the use of nocturnal noninvasive ventilation in a subset of the population. Our study found, however, a trend to a significant association between diurnal pCO2 and respiratory events, as well as an association between VC and respiratory events. These results may suggest that respiratory and sleep evaluation should be performed systematically in LGDM. The ATS consensus statement recommends annual evaluation for sleep-disordered breathing in patients with DMD starting from the time they become wheel chair bounded and/or when clinically indicated [30]. This recommendation may be applied to 2C and 2D LGMD.

### Prognosis and Mortality

Respiratory failure is a major cause of morbi-mortality in neuromuscular diseases [31] and was the main cause of death in DMD a few decades ago (up to 73% of patients in the study by Rideau *et al*., published in 1983) [4]. Identifying prognostic factors is essential in patients with neuromuscular disorders. However, no data exists to date for prognostic factors in 2C and 2D LGMD. A VC under 1000 ml, the presence of daytime hypercapnia and nocturnal hypoxemia have been previously described as mortality predictors in patients with Duchenne muscular dystrophy [32].

Left ventricular cardiac dysfunction has already been associated with mortality in 2I LGMD (23, 24). In our cohort, cardiac impairment was significantly associated with mortality. A LVEF <55% was associated with an increased mortality in 2C and 2D LGMD. On the other hand, respiratory defects were associated with respiratory events but not with mortality. This observation suggests that it could be possible to overcome acute respiratory failure episodes, which occurred in a relevant proportion of our patients, and thus reducing respiratory related mortality. This encouraging result could be explained by the efficacy of long-term respiratory support technics, which have already been shown to improve survival in other neuromuscular diseases [33,11,34,35]. Improved cardiology and respiratory management of neuromuscular disease patients has been associated with prolonged survival in DMD patients [36,37]. Kohler *et al*. [38] reported an 85% survival at 30 years in DMD patients. As a consequence of the improved respiratory management, cardiac failure has become the main cause of mortality in DMD. Bach *et al*. [39] reported 52% of cardiac mortality and 21% of respiratory mortality among patients with DMD. In our study, global mortality rate was 21% with half for primary cardiac causes, despite ACE inhibitors and beta-blocker regimens.

### Limitations of the Study

Our study was mono-centric and retrospective and the results may be hampered by the limited sample size and the recruitment specificities of our unit. Future multi-centric studies may be helpful to better characterize the phenotype and prognosis of type 2C and 2D LGMD.

## Conclusion

In our cohort of severely respiratory impaired LGMD 2C and 2D patients, we observed a 21% mortality rate. Significant respiratory events occurred in 38% of patients over a median follow-up of 6 years. Left ventricular dysfunction was observed in 38% of patients and was associated with mortality. Both cardiac failure and respiratory impairment seem to have an important prognostic impact on morbidity and mortality in LGMD 2C and 2D patients, but the evolution of these two systems seems not to be correlated. These results warrant the need for a multidisciplinary follow-up of LGMD patients, including cardiologists and pulmonologists. Based on our experience and on the presented data, we recommend a yearly respiratory function evaluation including a sleep study, as well as cardiac defects screening in order to introduce treatment for heart failure when LVEF declines.
